# Correlation Between Oncotype DX, PREDICT and the Nottingham Prognostic Index: Implications for the Management of Early Breast Cancer

**DOI:** 10.7759/cureus.7552

**Published:** 2020-04-06

**Authors:** Christopher Hillyar, Hirah Rizki, Omar Abbassi, Sascha Miles-Dua, Gillian Clayton, Tasha Gandamihardja, Simon Smith

**Affiliations:** 1 Barts and the London School of Medicine and Dentistry, Queen Mary University of London, London, GBR; 2 Chelmsford Breast Unit, Mid Essex Hospitals National Health Service (NHS) Trust, Broomfield, GBR; 3 Surgery, Mid Essex Hospitals National Health Service (NHS) Trust, Broomfield, GBR

**Keywords:** oncotype dx, npi, predict, breast cancer recurrence, adjuvant chemotherapy, breast cancer, er-positive, lymph node-negative, her2-negative

## Abstract

Introduction

Breast cancer remains the most common cancer diagnosis in the UK. The current clinical practice utilises two different types of modalities to estimate the prognosis, risk of recurrence and benefit from adjuvant chemotherapy treatment in patients with early breast cancer. The first set of modalities includes risk calculators based on clinicopathological features, e.g. PREDICT or the Nottingham Prognostic Index (NPI); the second includes genetic profiling of tumour tissue using Oncotype DX (ODX; Genomic Health, Redwood City, CA) testing. PREDICT, NPI and ODX stratify breast cancers into high-, intermediate- and low-risk categories to help guide adjuvant chemotherapy treatment decisions. This study compares PREDICT, NPI and ODX Recurrence Scores (RS), with the aim of assessing 1) the correlation between the RS for PREDICT, NPI and ODX and 2) whether early breast cancer patients are stratified into similar risk categories by all three modalities.

Methods

This retrospective study included early breast cancer patients treated at a National Health Service (NHS) hospital over a 12-month period (October 1, 2017 to September 30, 2018). Inclusion criteria: consecutive patients with estrogen receptor (ER)-positive, human epidermal growth factor receptor 2 (HER2)-negative and lymph node-negative breast cancer. All patients were discussed at the local multidisciplinary team (MDT) meeting and underwent ODX testing. Exclusion criteria: patients without ODX test scores; patients with an in-breast recurrence; patients who did not undergo a sentinel lymph node biopsy (SLNB); and patients with ductal carcinoma in situ (DCIS) only. NPI and PREDICT scores were calculated for each patient using online tools, and ODX data was obtained through Genomic Health and MDT records. Patients were risk-stratified into high, intermediate and low risk of recurrence groups based on their PREDICT, NPI and ODX scores. The thresholds for risk stratification were based on current practice, which is evidence-based. Correlations between PREDICT, NPI and ODX scores were analysed using Spearman's correlation coefficient.

Results

Forty-six patients (mean age: 56 years), with a total of 57 early breast cancers, underwent ODX testing. Risk categories generated by PREDICT very strongly correlated with NPI for all patients (r=0.92; P<0.0001). However, the RS generated by ODX testing only strongly correlated for patients with low-risk PREDICT scores (r=0.51; P=0.0134), while no correlation between RS and PREDICT was observed for patients with intermediate- or high-risk PREDICT scores (r=-0.0064; P=0.9767). Similar results were seen between NPI and RS. Overall, only 19/46 (41.3%) patients had an RS which corresponded to PREDICT risk category, while 18/46 (39.1%) patients had an RS that indicated a higher risk of recurrence than PREDICT, and 9/46 (19.6%) patients had an RS indicating a lower risk of recurrence than PREDICT. Similar results were found when comparing RS and NPI.

Conclusion

The risk of recurrence estimated by ODX in patients deemed low risk by PREDICT or NPI highly correlated, while no such correlation existed in patients with an estimated intermediate- or high-risk breast cancer. In PREDICT- or NPI-estimated intermediate- and high-risk patients, ODX provided valuable additional prognostic information to guide adjuvant treatment, while the potential avoidance of ODX testing in low-risk patients presents significant cost-savings.

## Introduction

Breast cancer remains the most common type of cancer in the UK, accounting for 15% of all new cancer diagnoses. Approximately 55,200 new breast cancers are diagnosed each year [1]. Unfortunately, the five-year relative survival for breast cancer in women in England is below the European average. Overall survival is shown to have been improved through the delivery of chemotherapy [2]. However, chemotherapy regimens are associated with higher costs as they are more resource-intensive [3]. In addition, chemotherapy is associated with toxic side effects including cardiac toxicity, neutropenic sepsis, myelosuppression and neuropathy [4].

The absolute benefit of chemotherapy is proportional to the absolute risk of recurrence. Improved information on individual patients' risk of recurrence (i.e. prognostic risk) and predicted response to chemotherapy (i.e. predictive benefit) may help target chemotherapy to patients who will receive the greatest benefits [5]. In current practice, there are two main types of modalities utilised to estimate an individual patient's risk of recurrence and prognosis. The first set of modalities are risk calculators and scoring systems, such as PREDICT and the Nottingham Prognostic Index (NPI). These clinicopathological scoring systems incorporate features such as age at diagnosis, lymph node status, tumour size, histological grade, lymphovascular invasion and hormone receptor expression. NPI generates a five- and 10-year survival score, while PREDICT produces a score that predicts benefit from chemotherapy treatment.

The second set of modalities includes genetic profiling such as Oncotype DX (ODX; Genomic Health, Redwood City, CA). This 21-gene assay generates a Recurrence Score (RS) that predicts the benefit from chemotherapy treatment in terms of a reduction in the 10-year risk of recurrence for a particular breast cancer. A 'low' RS indicates a low risk of cancer recurrence even without chemotherapy treatment, while a 'high' RS predicts recurrence and indicates the benefit of chemotherapy treatment. The use of ODX testing helps to inform adjuvant chemotherapy treatment decisions by identifying patients who will derive the greatest benefit from chemotherapy and those who will not, thereby preventing over-treatment, unnecessary exposure to the risk of side effects and high cost of chemotherapy treatment [5].

That said, ODX testing is also expensive and costs £2,580 per patient [6]. By comparison, online scoring systems such as PREDICT and NPI are easily available online and free of cost. Therefore, the judicious use of ODX testing is also paramount. PREDICT, NPI and ODX all provide an indication of the recurrence risk for individual patients with early breast cancer, based largely on estimations of risk of breast cancer recurrence from established data [7-9]. This study compares PREDICT, NPI and ODX RS with the aim of assessing 1) the correlation between the RS for PREDICT, NPI and ODX and 2) whether early breast cancer patients are stratified into similar risk categories by all three modalities.

## Materials and methods

Patient population

This study reviewed consecutive early breast cancer patients treated at our National Health Service (NHS) institution over a 12-month period (October 1, 2017 to September 30, 2018). Inclusion criteria were as follows: consecutive patients with early breast cancer, defined as estrogen receptor (ER)-positive, human epidermal growth factor receptor 2 (HER2)-negative and lymph node-negative breast cancer. All patients were assessed at the local multidisciplinary team (MDT) meeting for ODX testing. Exclusion criteria were as follows: patients without ODX test scores; patients with an in-breast recurrence; patients who did not undergo a sentinel lymph node biopsy (SLNB); and patients with ductal carcinoma in situ (DCIS) only.

Data collection

Patients were identified through MDT records. Data were collected on patient demographics and clinicopathological factors from electronic patient notes and histology reports. NPI and PREDICT scores were calculated for each patient using online tools [7,9]. For PREDICT, version 2.0 was utilised and third-generation chemotherapy was selected for all patients. A 10-year survival benefit from chemotherapy was used to risk-stratify patients. The RS for each patient was obtained through Genomic Health (Genomic Health, Redwood City, CA).

Data analysis

Patients were risk-stratified into recurrence groups based on their chemotherapy benefits, prognosis and risk of RS generated by PREDICT, NPI and ODX, respectively [7-9]. Thresholds for risk stratification were based on current practice at our institution, which is evidence-based and is outlined in Table 1. Scatter plots for correlations between RS, PREDICT and NPI were analysed using Spearman’s correlation coefficient. Statistical analysis was performed using GraphPad Prism 8.1 (GraphPad Software, San Diego, CA).

**Table 1:** Thresholds used to risk-stratify patients based on risk of recurrence from Oncotype DX, PREDICT and Nottingham Prognostic Index RS: Recurrence Score generated through Oncotype DX testing; NPI: Nottingham Prognostic Index

RS	PREDICT	NPI
0–17, low risk	<3, low risk	≤2.4, excellent prognosis
		2.41–3.4, good prognosis
18–25, intermediate risk	3–5, intermediate risk	3.41–5.4, moderate prognosis
≥26, high risk	>5, high risk	≥5.41, poor prognosis

## Results

Patient and tumour characteristics

This study included 46 consecutive patients (mean age: 56 years) who underwent ODX genetic profiling at our institution over the study period. These patients were treated for 57 ER-positive, HER2-negative and lymph node-negative early breast cancer. Table 2 summarises patient and tumour characteristics. The median tumour size was 2.4 cm. The median tumour grade was 3; 14/46 (30%) patients had screen-detected tumours, and 8/46 (30.4%) had multifocal disease. The median PREDICT, NPI and RS were 3, 3.4, and 17, respectively.

**Table 2:** Patient and tumour characteristics RS: Recurrence Score generated through Oncotype DX testing; NPI: Nottingham Prognostic Index

	Number	%	Mean/median (range)
Total tumours	57	-	-
Tumour size (cm), median (range)	-	-	2.4 (0.4–8.5)
Grade 1	2	3.5	-
Grade 2	26	45.6	-
Grade 3	29	50.8	-
Total patients	46	-	-
Patient age, mean (range)	-	-	56 (35–75)
Cancer identified by symptomatic presentation	32	69.6	-
Cancer identified through screening	14	30.4	-
Patients with single tumours	38	82.6	-
Patients with multifocal disease	8	17.4	-
PREDICT score, median (range)	-	-	3 (1–6)
Low (<3)	22	47.8	
Intermediate (3-5)	22	47.8	
High (>5)	2	4.3	
NPI score, median (range)	-	-	3.4 (1.84–4.58)
Excellent (≤2.4)	6	13	-
Good (2.41–3.4)	21	45.7	-
Moderate (3.41–5.4)	19	41.3	-
Poor (≥5.41)	0	0.0	-
RS, median (range)	-	-	1.7 (0–82)
Low (<18)	24	52.2	-
Intermediate (18-25)	7	15.2	-
High (>25)	15	32.6	-

Correlation between PREDICT and Nottingham Prognostic Index

**Figure 1:**
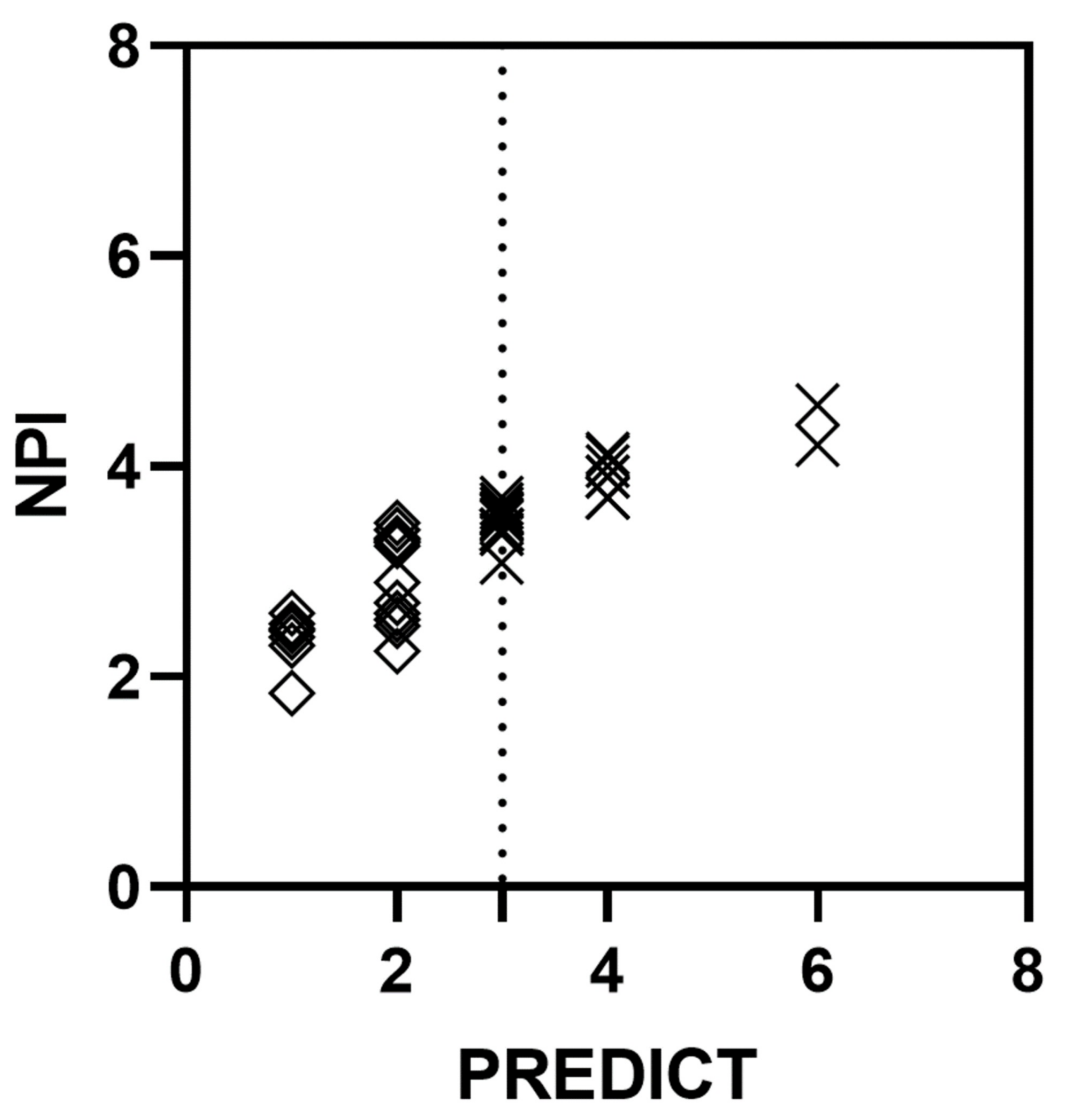
Correlation between PREDICT and NPI for early breast cancer Correlation between PREDICT and NPI for all patients, Spearman r=0.92 (P<0.0001); dotted line: threshold for PREDICT score between low (<3) and intermediate/high (≥3); correlation between PREDICT and NPI for patients with low PREDICT score (diamonds), Spearman r=0.66 (P=0.0009); correlation between PREDICT and NPI for patients with intermediate/high PREDICT score (crosses), Spearman r=0.86 (P<0.0001) NPI: Nottingham Prognostic Index

Figure 1 shows a comparison of NPI and PREDICT for all patients included in this study. A Spearman r test demonstrated a highly significant and very strong overall correlation between the PREDICT calculated risk of recurrence compared to the score generated through NPI (all patients: r=0.92; P=0.0001). A sub-analysis stratified by risk category demonstrated that PREDICT correlated strongly with NPI for patients with both low PREDICT score (i.e. PREDICT score of <3) and intermediate/high PREDICT score (i.e. PREDICT score of ≥3; r=0.66, P=0.0009 and r=0.86, P=0.0001, respectively).

Correlation between PREDICT and Oncotype DX

**Figure 2:**
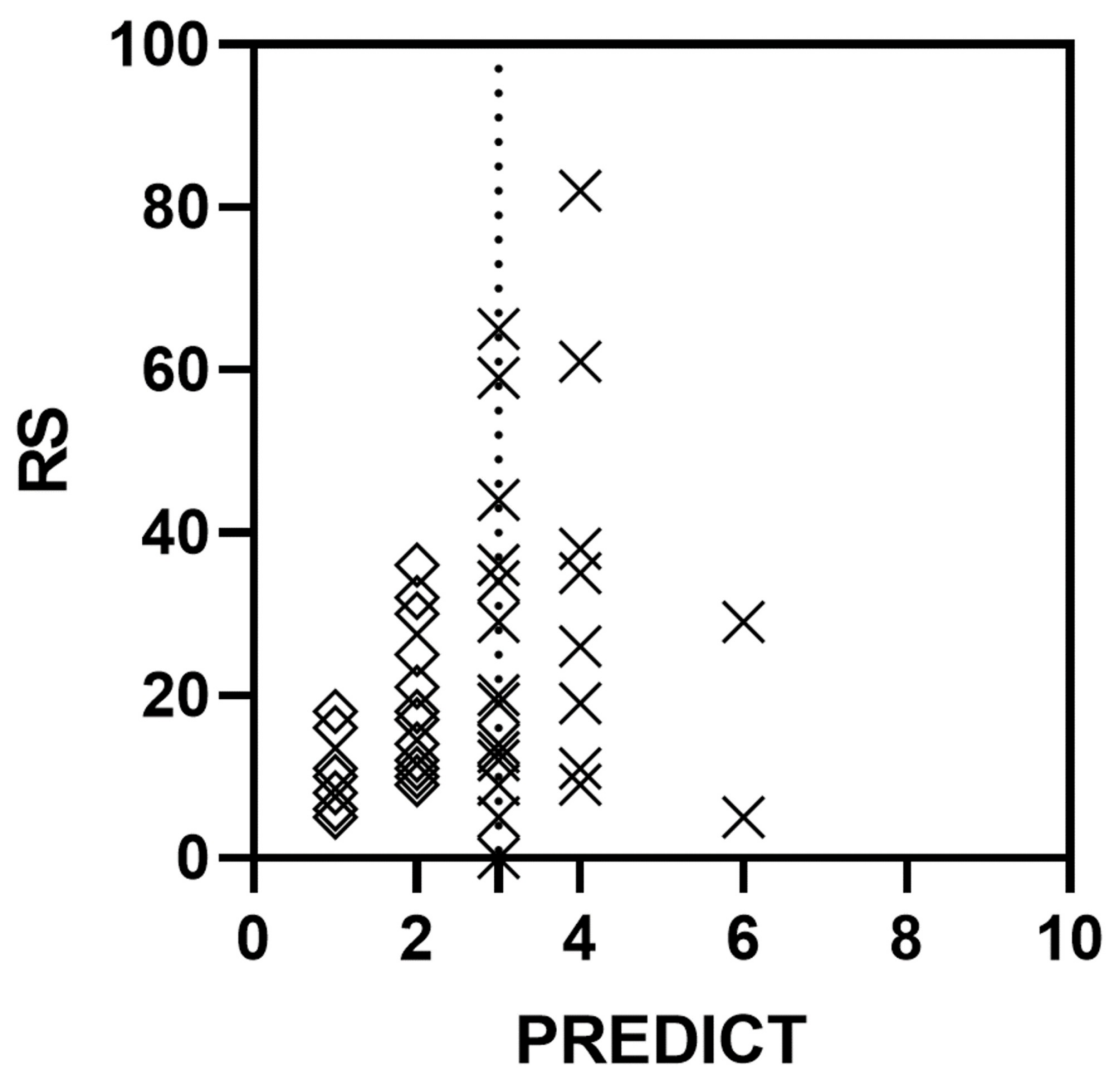
Correlation between PREDICT and ODX in early breast cancer Correlation between PREDICT score and the RS from ODX testing for all patients, Spearman r=0.34 (P<0.0193); dotted line: threshold for PREDICT score between low (<3) and intermediate/high (≥3); correlation between PREDICT and RS for patients with low PREDICT score (diamonds), Spearman r=0.51 (P=0.0134); correlation between PREDICT and RS for patients with intermediate/high PREDICT score (crosses), Spearman r=-0.0064 (P=0.9767) RS: Recurrence Score generated through Oncotype DX testing; ODX: Oncotype DX

**Figure 3:**
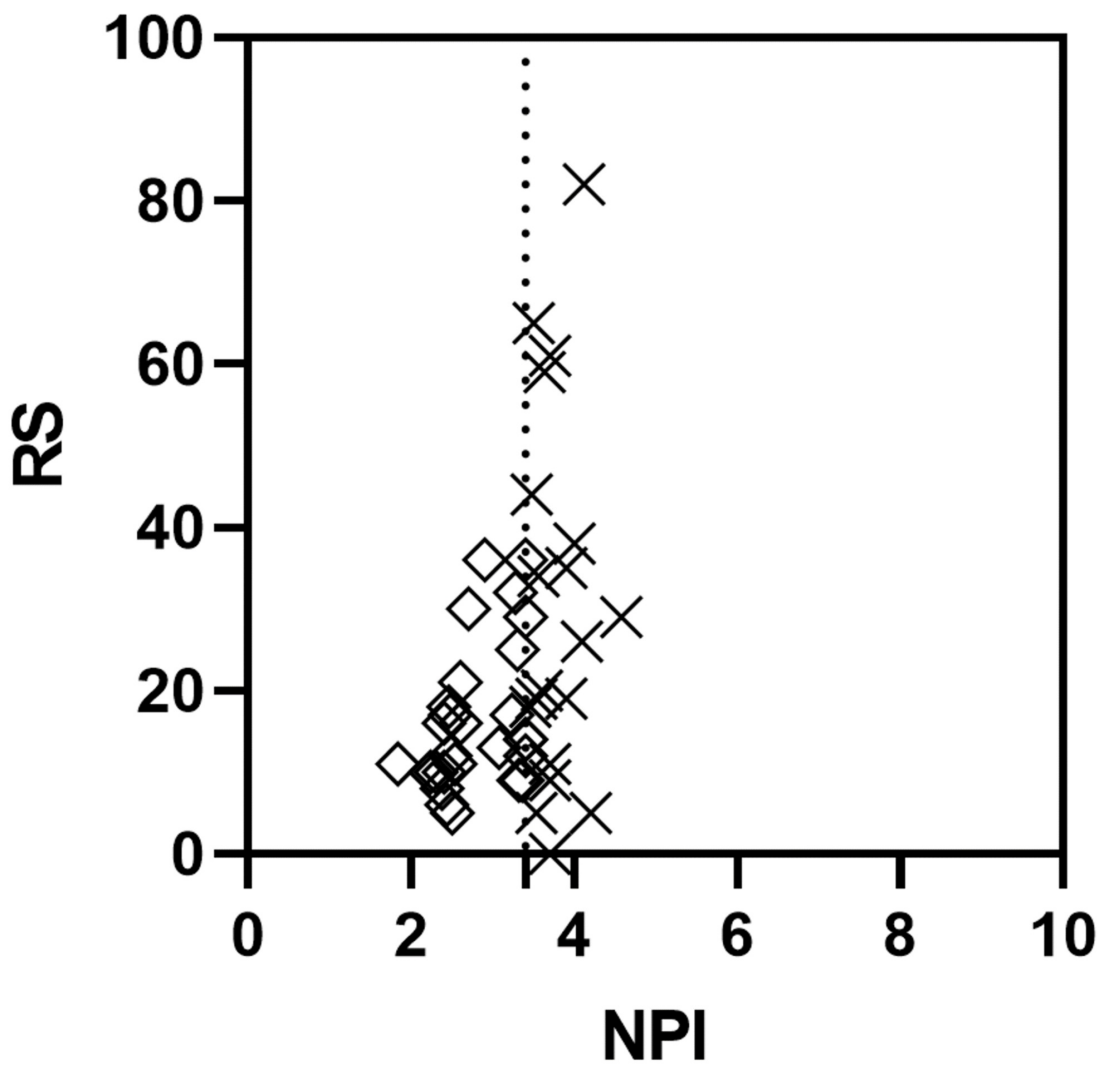
Correlation between Nottingham Prognostic Index and ODX in early breast cancer Correlation between NPI and RS from ODX testing for all patients, Spearman r=0.37 (P=0.0099); dotted line: threshold for NPI between excellent/good (≤3.4) and moderate/poor (>3.4); correlation between NPI score and RS for patients with excellent/good NPI (diamonds), Spearman r=0.42 (P=0.0302); correlation between NPI and RS for patients with moderate/poor Nottingham Prognostic Index score (crosses), Spearman r=0.0093 (P=0.9700) RS: Recurrence Score generated through Oncotype DX testing; NPI: Nottingham Prognostic Index; ODX: Oncotype DX

Overall, the PREDICT score and the RS from ODX were only weakly, albeit significantly, correlated when scores for all patients were analysed (r=0.34, P=0.0193; Figure 2). A similarly weak, yet significant correlation was also found between NPI and RS for all patients (r=0.38, P=0.0099). However, a sub-analysis of patients with low PREDICT score (i.e. PREDICT score of <3) demonstrated a strong and significant correlation between PREDICT and RS (r=0.51, P=0.0134), while the correlation between PREDICT and RS was very weak and non-significant for patients with intermediate/high PREDICT score (i.e. PREDICT score of ≥3; r=-0.0064, P=<0.9767). Similar results were observed when analysing the strength and significance of a correlation between NPI and RS (patients with excellent/good risk NPI score of ≤3.4: r=0.42, P=0.0302; and patients with moderate/poor risk NPI score of >3.4: r=0.0093, P=0.9700; Figure 3). Together, this suggested that patients with an estimated low risk of recurrence using PREDICT or NPI are likely to receive a similarly low recurrence risk from ODX testing.

The degree of disparity between PREDICT score and RS is shown below (Table 3). It shows the stratification of patients by PREDICT and RS risk category; 7/46 (15.2%) patients had a low PREDICT score but either an intermediate or high RS, and 9/46 (19.6%) patients had either an intermediate or high PREDICT score but low RS. Similar results were also observed when comparing NPI and RS, suggesting that the majority of patients (58.7%) were assigned to different categories of recurrence risk depending on whether this was based on the PREDICT/NPI or RS (Table 4).

**Table 3:** Stratification of early breast cancer patients by PREDICT and Oncotype DX categorical scores RS: Recurrence Score generated through Oncotype DX testing

	RS (N = 46)		
PREDICT risk category (N = 46)	Low (n = 24)	Intermediate (n = 7)	High (n = 15)
Low (n = 22), n (%)	15 (32.6)	4 (8.7)	3 (6.5)
Intermediate (n = 22), n (%)	8 (17.4)	3 (6.5)	11 (23.9)
High (n = 2), n (%)	1 (2.2)	0 (0)	1 (2.2)

**Table 4:** Stratification of early breast cancer patients by Nottingham Prognostic Index and Oncotype DX categorical scores RS: Recurrence Score generated through Oncotype DX testing; NPI: Nottingham Prognostic Index

	RS risk category (N = 46)		
NPI risk category (N = 46)	Low (n = 24)	Intermediate (n = 7)	High (n = 15)
Excellent (n = 6)	6	0	0
Good (n = 21)	13	3	5
Moderate (n = 19)	5	4	10
Poor (n = 0)	0	0	0

In total, only 19/46 (41.3%) patients had an RS risk category that corresponded with their PREDICT risk category (Table 5). In fact, 18/46 (39.1%) patients had a higher RS risk category compared to their PREDICT risk category, while 9/46 (9.1%) patients had a lower RS risk category compared to their PREDICT risk category. Again, similar results were also found when comparing NPI and RS risk categories, suggesting that, for a majority of patients, the risk categories were changed after ODX testing (Table 6).

**Table 5:** Changes in PREDICT-assigned risk category after Oncotype DX testing in early breast cancer RS: Recurrence Score generated through Oncotype DX testing

	Change in risk category		
	RS risk category lower than PREDICT risk category	RS risk category equivalent to PREDICT risk category	RS risk category higher than PREDICT risk category
Patients, n (%)	9 (19.6)	19 (41.3)	18 (39.1)

**Table 6:** Changes in Nottingham Prognostic Index-assigned risk category after Oncotype DX testing in early breast cancer RS: Recurrence Score generated through Oncotype DX testing; NPI, Nottingham Prognostic Index

	Change in risk category		
	RS risk category lower than NPI risk category	RS risk category equivalent to NPI risk category	RS risk category higher than NPI risk category
Patients, n (%)	8 (17.4)	20 (43.5)	18 (39.1)

## Discussion

NPI and PREDICT are tools commonly used in the UK by MDTs to estimate the risk of recurrence to guide adjuvant chemotherapy treatment in early breast cancer. This study demonstrates that the risk of recurrence estimated by PREDICT strongly correlated with that generated by NPI. Therefore, either modality is likely to generate a similarly low, intermediate or high risk of recurrence for a given patient. However, several studies have highlighted the shortcomings and questioned the validity of NPI and PREDICT [10-12]. A study by Plakhins et al. showed that overall survival and breast cancer-specific survival obtained from PREDICT were significantly lower than the observed survival of the study population [12]. Lambertini et al. have shown NPI to also significantly underestimate 10-year overall survival in both young and old patients [13]. This is reflected in our results, which demonstrated that nearly 40% of the patients had a higher risk of recurrence after ODX testing than that based initially on PREDICT or NPI.

There were three patients in our study who had a PREDICT 10-year benefit from adjuvant chemotherapy of <3% (low risk); however, after ODX testing, these three patients had an RS of ≥26 (high risk). These patients were between 47-67 years of age with grade 2-3 unifocal cancers, measuring 14-45 mm in size. One patient in our study had a PREDICT 10-year benefit from adjuvant chemotherapy of >5% (high risk); however, after ODX testing, this patient had an RS of ≤17 (low risk). This patient was 35 years of age with grade 3 unifocal cancer, measuring 60 mm in size.

A recent systematic review has suggested that ODX testing is superior to online prognostication tools at assessing recurrence risk [14]. NPI was developed in 1982 and PREDICT was validated by Wishart et al. in 2010 [8,9]. Breast cancer is recognised as a heterogeneous, phenotypically diverse disease with distinct behaviours and responses to therapy, and, therefore, these scoring systems provide little information regarding how individual cancers will respond to treatment [14]. This is where the advent of genetic profiling, such as ODX and Prosignia (NanoString Technologies, Seattle, WA), is playing a significant role in guiding adjuvant chemotherapy treatment.

In our study, the majority of patients estimated to be at a low risk of recurrence by NPI/PREDICT were also found to have a low RS. The correlation between these scoring modalities was statistically significant and strong in the sub-analysis of patients with low NPI/PREDICT score, suggesting concordance between these three risk predictive modalities for low-risk patients. However, nearly 60% of all patients, irrespective of NPI/PREDICT score, had a PREDICT or NPI risk category that was different from the risk category estimated through ODX testing. In the subgroup of patients with PREDICT/NPI intermediate or high risk, the correlation between the recurrence risk generated by RS was very weak and non-significant. This has implications for adjuvant treatment. A recent systematic review and meta-analysis have demonstrated that ODX testing significantly changed the clinicopathologically based adjuvant chemotherapy recommendations [15].

The landmark TAILORx Trial advocated the use of ODX to guide adjuvant treatment [7]. This 21-gene assay may identify up to 85% of women with early breast cancer, who can be spared adjuvant chemotherapy, especially those who are older than 50 years in age and have an RS of 25 or lower [7]. ODX was found to be a more accurate predictor of relapse than standard clinical features for individual patients [16]. Furthermore, ODX testing has been shown to result in cost-savings and a significant reduction in chemotherapy administration [7,17]. A meta-analysis by Carlson et al. reported that ODX testing changed treatment decisions for 49% of the patients [15]. Similarly, a study by Smyth et al. has noted that 59% of treatment recommendations changed after ODX testing [17].

At present, the National Institute for Health and Care Excellence (NICE) recommends the use of ODX testing to guide the decision-making on chemotherapy after surgery in ER-positive, HER2-negative and lymph node-negative early breast cancer patients [18]. In particular, NICE advocates the utilisation of ODX only for patients deemed to be at an intermediate risk of recurrence. Indeterminate risk is defined by NICE as an NPI of greater than 3.4, or patients identified as intermediate risk by another validated scoring system (e.g. PREDICT). In support of the NICE guidance, this study demonstrates that for intermediate-risk patients, there is a lack of correlation between PREDICT/NPI and RS, thus justifying the use of ODX not only in this cohort but also for PREDICT/NPI high-risk patients as well.

A potential limitation of this study is the fact that the dataset may have under-represented ethnic minorities and young patients. In addition, the cohort included in this study was relatively small, limiting the generalisability of our conclusions.

## Conclusions

In conclusion, the results of this study have two implications for clinical management. First, patients with low-risk PREDICT or NPI scores are unlikely to have their management altered by ODX testing since low-risk PREDICT and NPI scores and RS are strongly correlated; therefore, this represents an opportunity for cost-saving by avoiding unnecessary and expensive genetic testing in patients for whom the results of ODX testing may provide no additional information over that offered by PREDICT or NPI. Secondly, patients with intermediate-/high-risk PREDICT or NPI scores are more likely to receive an RS that places them in a different risk category with regards to recurrence risk and, hence, the RS may be utilised by the MDT to guide adjuvant treatment decisions in patients with intermediate-/high-risk PREDICT or NPI scores. Further research is required to see if these results could be extended to larger and more diverse patient populations.
